# Numerical Investigation of Enhanced Heat Transfer with Micro Pin Fins in Heat Exchangers

**DOI:** 10.3390/mi15091120

**Published:** 2024-08-31

**Authors:** Qin Zhou, Hongyan Wang, Fuyuan Wu, Shengfei Liu, Huafeng Wei, Guoqing Hu

**Affiliations:** 1 Department of Engineering Mechanics, State Key Laboratory of Fluid Power and Mechatronic Systems, Zhejiang University, Hangzhou 310027, China; zhouqin2020@zju.edu.cn (Q.Z.); 12224044@zju.edu.cn (S.L.); 2 Zhejiang Kangsheng Co., Ltd., Hangzhou 311700, China; wanghy@kasun.cn (H.W.); wufy@kasun.cn (F.W.)

**Keywords:** micro pin fin, pin pitch, pin diameter, heat transfer enhancement, heat exchanger

## Abstract

Pin-fin and flat-tube heat exchangers (PFFTHXs) offer a promising alternative to traditional louvered-fin and flat-tube heat exchangers (LFFTHXs), especially when used as evaporators. The streamlined structure of pin fins helps to effectively remove condensate and defrost water. In this study, we conducted a numerical analysis of 60 different pin-fin configurations across three pin diameters to enhance heat transfer in PFFTHXs. Our investigation focused on how pin pitch affects both airflow and heat transfer efficiency. The results show that a closer pin pitch increases both the heat transfer rate per unit area and the pressure drop for a given airflow velocity. We evaluated the overall performance of these configurations using the heat transfer rate per unit frontal area obtained at equivalent fan power levels. The analysis identified optimal configurations for each pin diameter, with the 0.2 mm diameter configuration demonstrating the highest heat transfer efficiency—this was on par with louvered fins but used fewer resources. This makes it an ideal choice for evaporative applications in PFFTHXs.

## 1. Introduction

Improving the performance of heat exchangers has become increasingly important due to rising energy demands in both the industrial and residential sectors. Louvered-fin and flat-tube heat exchangers (LFFTHXs) are preferred for their compact design, low refrigerant consumption, and robustness under high pressure, making them ideal for air conditioning, heat pumps, and refrigeration systems. However, their complex fin structure compromises drainage efficiency when used as evaporators, leading to moisture-related problems [1] and increasing energy consumption while reducing comfort during frost conditions [2]. Alternatively, pin-fin and flat-tube heat exchangers (PFFTHXs) offer a streamlined fin design that improves the drainage of condensate and defrost water, making them a superior choice for evaporative applications.

Pin fins are known for their exceptional heat transfer capabilities due to their ability to disrupt the boundary layer—a feature that has been exploited in various high-performance applications. Most notably, they are used in turbine blade cooling. Effendy et al. [3] designed elliptical pin fins strategically oriented to optimize airflow for the improved trailing-edge cooling of turbine blades, effectively balancing high heat transfer with a reduced pressure drop. Xie et al. [4] have also demonstrated the significant benefits of pin fins in improving heat transfer at turbine blade tips. Outside of aerospace, pin fins have been instrumental in electronics cooling. Yan et al. [5] introduced an innovative micro-pin-fin heat sink with fin-shaped strips that improved heat dissipation for electronic chips by creating greater flow disturbance. Similarly, Markowski et al. [6] found that pin-fin heat sinks outperformed conventional plate-fin designs under natural convection conditions. In addition, pin fins have been shown to be effective in solar air heaters [7,8] and in harnessing heat from automotive exhaust systems [9], further validating their broad utility and effectiveness in advanced thermal management applications.

Advances in manufacturing processes have spurred numerous studies aimed at improving heat transfer in gas-to-liquid heat exchangers via pin-fin structures (also referred to as wire structures in some sources). Arie et al. [10,11] studied two additively manufactured cross-media polymer-composite heat exchangers of different sizes. These exchangers, with cores of continuous metal wires and polymer tubes, demonstrated superior heat transfer rates per unit mass compared to traditional louvered-fin, wavy-fin, and plain-plate-fin heat exchangers. Fugmann et al. [12] established a test rig to evaluate the heat transfer capabilities of micro pin fins in PFFTHXs and found that a 0.25 mm diameter pin fin doubled the heat transfer coefficient of a standard louver fin. In addition, Sahiti [13] developed a method to optimize the height of pin fins in PFFTHXs by modifying fin parameters, achieving optimal heights for different pin-fin configurations.

The configuration of the pin fins significantly affects the effectiveness of heat transfer enhancement [14,15]. Pandit et al. [9] conducted an experimental investigation on the heat transfer enhancement provided by diamond-shaped pin fins in rectangular channels. They found that a ratio of pin-fin height to channel height of 0.5 gave the optimum heat transfer performance. Xu et al. [16] conducted experiments on six different sets of pin fins in a wide channel and found that circular pin fins exhibited the highest heat transfer capability. A study by Choudhary et al. [17] on cylindrical pin fins showed that pin pitch significantly affects both heat transfer and friction loss.

Numerical simulations have become essential for investigating the effect of pin-fin geometry on heat transfer enhancement, particularly due to the high cost and challenges associated with experimental studies. Narato et al. [18] used computational fluid dynamics (CFD) simulations to study the influence of pin inclination angles within arrays in a rectangular channel and found that jet-like flows behind inclined pins played a significant role in heat transfer enhancement. Kishore et al. [19,20] performed numerical analysis on pin-fin arrays optimized by the particle swarm optimization algorithm and found that the piranha pin fin excelled in heat transfer efficiency. Zhang et al. [21] investigated the effect of pin-fin shape on the performance of arrays in a rectangular channel, emphasizing the importance of considering thermal loading conditions when selecting the optimal configuration. Other numerical studies have investigated the effects of pin pitch, including both streamwise and transverse pitch, on heat transfer enhancement. Li et al. [22] analyzed different pin-fin configurations in a rectangular mini-channel and found that the heat transfer capacity did not vary monotonically with the streamwise pitch. Fugmann et al. [23,24] performed two-dimensional simulations on 0.1 mm diameter pin-fin structures in PFFTHXs and observed increased heat transfer and flow resistance in configurations with smaller transverse pitches. A similar phenomenon was reported by Jin et al. [25] in their study of forced convective heat transfer through pin-fin arrays in a rectangular channel. However, these important studies focused exclusively on a single pin diameter. The flow field and thermal performance of micro pin fins in PFFTHXs, as influenced by different pin diameters and pitches, remain areas for future investigation.

In this study, we used CFD simulations to investigate the heat transfer enhancement of different pin-fin configurations in PFFTHXs. We comprehensively analyzed the effect of streamwise and transverse pin pitches on the flow and heat transfer characteristics within micro-pin-fin arrays, considering three different pin diameters in 60 different configurations. Optimal pin-fin configurations were identified for each diameter and then compared to conventional louvered fins. Based on these comparisons, we recommend a specific micro-pin-fin configuration that demonstrates satisfactory heat transfer efficiency for application in PFFTHXs as evaporators.

## 2. Geometrical and Numerical Models

**Figure 1:**
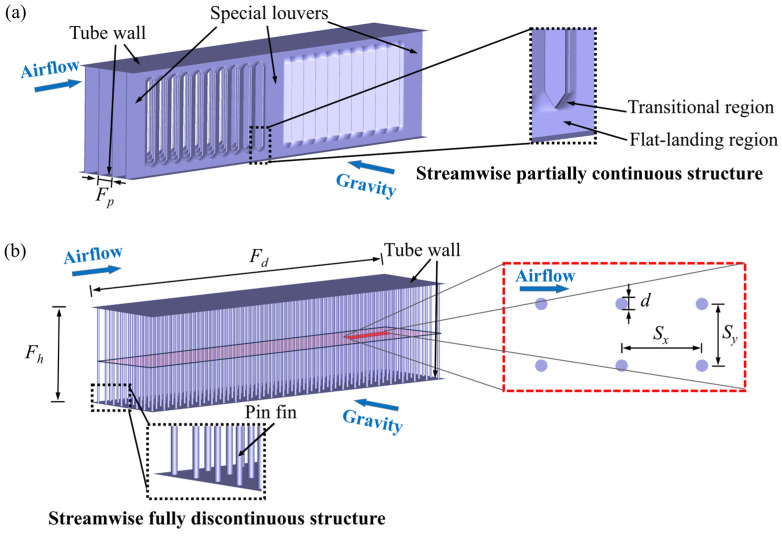
Schematics of flat-tube heat exchangers with different fins: (**a**) louvered fin; (**b**) pin fin.

As depicted in Figure 1a, louvered fins have several complex structures, including special louvers, a transitional region, and a flat-landing region, which ensure that the structure remains continuous in the streamwise direction. However, they are unfavorable for drainage as they hinder the movement of accumulated water within the LFFTHXs of vertical flat tubes and horizontal fins. Two conventional louvered fins are chosen to evaluate the heat transfer efficiency of pin fins, which have fin pitches *F_p_* of 1.2 mm and 1.4 mm, respectively, with the other geometric parameters remaining the same—they are named LF-1 and LF-2, respectively. For the sake of brevity, only the geometrical and numerical models of PFFTHXs are introduced in this section, and the details for the LFFTHXs can be found in our previous work [26].

### 2.1. Geometrical Model

The schematic of PFFTHXs is shown in Figure 1b. Considering the challenges in manufacturing micro pin fins, our study concentrates solely on the circular pin fin. The micro-pin-fin array consists of a series of regularly arranged aluminum cylinders. The fin structure, which is fully discontinuous and uniform in the streamwise direction, contributes to draining the accumulated water within the array out of the heat exchanger. The fin height *F_h_* and flow depth *F_d_* are kept at the same value as the selected louvered fins, with values of 8 mm and 32 mm, respectively. Three groups of micro pin fins with different pin diameters *d* are selected in our simulations, with 60 configurations in total. In order to obtain configurations with superior overall performance, the transverse pin pitches, *S_y_*, for these three sets of fins are chosen to be different values to fit the variation in pin diameters, whereas the streamwise pin pitches, *S_x_*, are maintained at the same values due to their significant effect on the drainage performance, with the minimum value greater than the width of the louver gaps. The detailed geometric parameters of the pin fins are listed in Table 1.

### 2.2. Governing Equations

In our simulations, the flat tube is treated as a constant temperature wall, consistent with the assumptions used in some previous work [13,26,27,28,29] on flat-tube heat exchangers. The airflow is assumed to be a steady-state incompressible flow. According to the experiments of Bergelin et al. [30] on tube banks, the flow is predominantly laminar for Reynolds numbers less than 200. For the diameters and velocities studied in this paper, the maximum Reynolds number is 135.2, and thus the airflow is considered as laminar in the calculations, given that the flow through the pin fins is similar to that through the tube banks.

The mass, momentum, and energy conservation equations for the airflow are as follows:

(1) $$\nabla \cdot (\rho_{a}u)=0$$

(2) $$\nabla \cdot \left(\rho_{a}uu\right)=-\nabla p+\nabla \cdot (\mu_{a}\nabla u)$$

(3) $$\nabla \cdot \left(\rho_{a}c_{p,a}uT\right)=\nabla \cdot \left(k_{a}\nabla T\right)$$

The energy equation for the fin domain is given as

(4) $$\nabla \cdot \left(k_{f}\nabla T\right)=0$$

where ***u***, *ρ*, *p*, *T*, *μ*, *k*, and *c_p_* are the velocity, density, pressure, temperature, dynamic viscosity, thermal conductivity, and specific heat, respectively, with the subscripts “*a*” and “*f*” indicating the air and fin regions, respectively.

### 2.3. Computational Domain and Boundary Conditions

**Figure 2:**
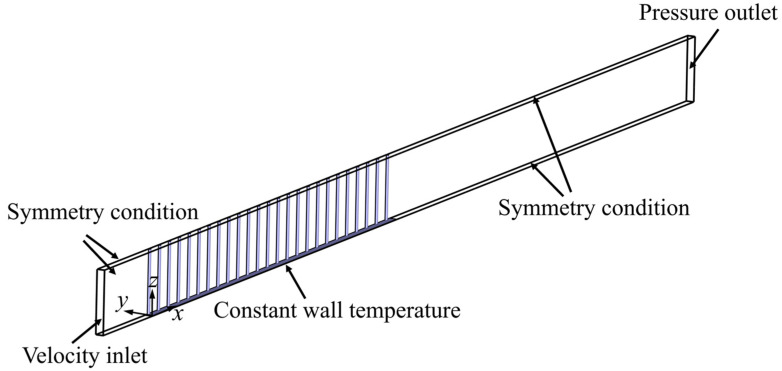
Three-dimensional computational domain and boundary conditions for CFD analysis.

Taking advantage of the symmetry in the fin height and transverse directions, CFD analysis of the airflow and micro-pin-fin arrays is performed only for half the fin height (*F_h_*/2) and half the transverse pin pitch (*S_y_*/2), consistent with the numerical studies of the conjugate heat transfer between the micro-pin-fin structures and air by Sahiti [13], Fugmann et al. [31], and Kailkhura et al. [32]. As shown in Figure 2, the computational domain is extended both upstream and downstream to avoid flow oscillations and backflow at the inlet and outlet. Since the inlet effect is less significant, the upstream extension is shorter than the downstream extension, with lengths of 0.8 *F_h_* and 4.9 *F_h_*, respectively. *x*, *y*, and *z* coordinates indicate the streamwise direction of the fin array from the leading edge of the tube wall, the transverse direction of the fin array from the midplane between transverse adjacent pins, and the height direction of the fins from the bottom tube wall, respectively. The velocity inlet boundary condition and pressure outlet boundary condition are applied at the inlet and outlet of the computational domain, respectively, with the inlet air temperature *T_in_* and the outlet gauge pressure *p_out_* maintained at 290.15 K and 0 Pa, respectively. A no-slip boundary condition with a constant temperature is used for the tube wall, and the wall temperature *T_w_* is set to 274 K. All other external boundaries are symmetric planes, and the air-fin interface satisfies the no-slip boundary condition with conjugate heat transfer. Note that in Section 3.1, we mirror the flow and temperature fields along the transverse symmetry plane during part of the post-processing to intuitively demonstrate the flow pattern and heat transfer characteristics within pin-fin arrays. The thermophysical properties of air and aluminum pin fin are given in Table 2.

### 2.4. Solution Methods

The finite volume method-based software ANSYS Fluent 19.2 is used to analyze the conjugate heat transfer interaction between airflow and pin-fin arrays. The SIMPLE algorithm is applied for the pressure–velocity coupling. The convection terms of the momentum and energy equations are discretized using a second-order upwind scheme, while the pressure term is discretized using a second-order central differencing scheme. Under-relaxation factors are set to 0.3, 0.7, and 1.0 for pressure, momentum, and energy, respectively. The residuals for the convergence criteria of the continuity, momentum, and energy equations are set to be 10^−4^, 10^−6^, and 10^−8^, respectively.

### 2.5. Parameter Definitions

In this study, the flow conditions are characterized by the Reynolds number *Re*, defined as

(5) $$Re=\frac{\rho_{a}U_{c}d}{\mu_{a}}$$

where the characteristic velocity *U_c_*, corresponding to the airflow velocity at the cross-section with minimal flow area, can be obtained from the inlet velocity *U_in_* as

(6) $$U_{c}=\frac{U_{in}A_{in}}{A_{c}}$$

The ratio of the frontal area, *A_in_*, to the minimal flow area, *A_c_*, can be obtained by

(7) $$\frac{A_{in}}{A_{c}}=\frac{S_{y}}{S_{y}-d}$$

The heat transfer coefficient, *h*, and the heat transfer rate, *Q*, are calculated from

(8) $$h=\frac{Q}{A_{o}\Delta T_{m}}$$

(9) $$Q=\rho_{a}U_{in}A_{in}c_{p,a}(T_{in}-T_{out})$$

where *A_o_* and *T_out_* are the total heat transfer area and outlet temperature, respectively, and the logarithmic mean temperature difference, Δ*T_m_*, is given as

(10) $$\Delta T_{m}=\frac{T_{in}-T_{out}}{\ln(\frac{T_{in}-T_{w}}{T_{out}-T_{w}})}$$

The pressure drop between the inlet and outlet, Δ*p*, is calculated as

(11) $$\Delta p=p_{in}-p_{out}$$

Considering the differences in the transverse pitches between the configurations, the heat transfer rate per unit frontal area, $\overset{-}{Q}$, is selected for analysis and given as

(12) $$\bar{Q}=\frac{Q}{A_{in}}$$

To evaluate the overall performance of the different configurations, the fan power per unit frontal area consumed by the heat exchanger, $\overset{-}{P}$, is chosen and determined by

(13) $$\bar{P}=\Delta pU_{in}$$

Furthermore, the pressure difference between the leading and trailing edges of an individual pin, *dp*, is selected to study the evolution of the flow structure along the streamwise direction and obtained by

(14) $$dp_{N}=p_{N}^{L}-p_{N}^{T}$$

where *p^L^* and *p^T^* are the pressures at the leading and trailing edges of the pin, respectively, and the subscript “*N*” indicates the pin at the *N*th position in the streamwise direction.

### 2.6. Grid Independence Study

**Figure 3:**
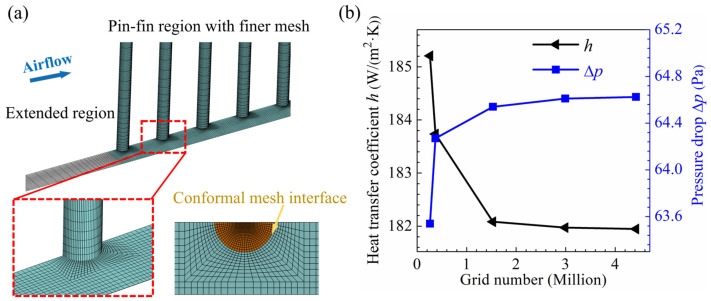
Grid details and grid independence study: (**a**) mesh configuration in the pin-fin region and extended region; (**b**) variation of heat transfer coefficient and pressure drop with the grid number for pin fin at *U_in_* = 3 m/s (*d* = 0.2 mm, *S_x_* = 0.7 mm, and *S_y_* = 0.8 mm).

In order to improve the computational accuracy and efficiency, hexahedral elements are used to discretize the computational domain, with a finer mesh in the pin-fin region. A high-resolution conformal mesh is applied at the air-fin interface to enhance the interpolation accuracy, as shown in Figure 3a. The pin fin with *d* = 0.2 mm, *S_x_* = 0.7 mm, and *S_y_* = 0.8 mm is chosen for the grid independence study at four velocities (*U_in_* = 1.5 m/s, 2 m/s, 2.5 m/s, and 3 m/s). Five meshes with different resolutions are selected, and the numbers of their elements are 0.26 million, 0.37 million, 1.53 million, 2.99 million, and 4.42 million, respectively, with the sizes of the first layer elements on the fin surface, Δ*s*, being *d*/8, *d*/10, *d*/25, *d*/40, and *d*/55, respectively. At all velocities, the variation in the heat transfer coefficient and pressure drop decreases with more elements, as shown in Figure 3b for the case of *U_in_* = 3 m/s. With an increase in the grid number from 1.53 to 4.42 million, the maximum differences in the heat transfer coefficient and pressure drop for the four velocities are 0.07% and 0.23%, respectively, indicating that the grid resolution with Δ*s* = *d*/25 provides a reasonable balance between accuracy and cost, which is used in subsequent simulations. For the 60 configurations of pin fins analyzed, a total of 480 simulations are performed to comprehensively investigate the effects of the pin pitches on the flow and heat transfer characteristics of pin-fin arrays, with a maximum grid number of 3.31 million.

### 2.7. Model Validation

**Figure 4:**
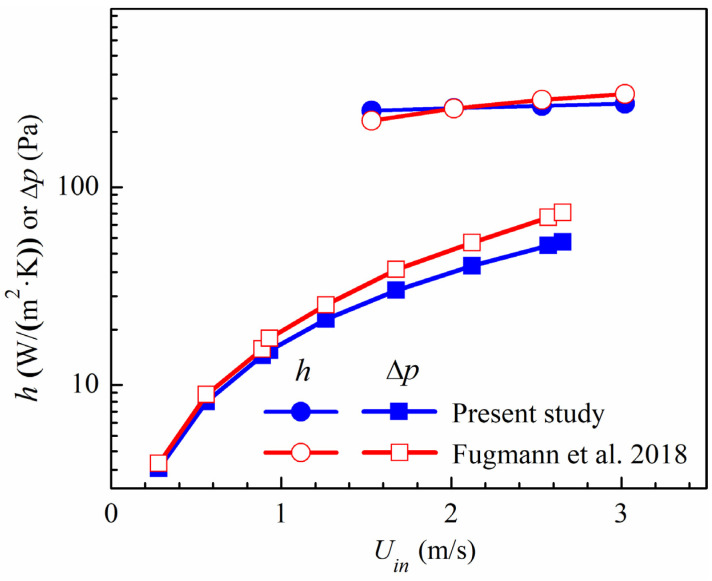
Comparison of numerical results with experimental data of Fugmann et al. [12].

The reliability of the numerical simulations for louvered fins has been confirmed in our previous work [26]. In this section, we focus on validating the numerical simulations for the pin fins. The experimental study on micro pin fins by Fugmann et al. [12] was used to validate the accuracy of our numerical method. Figure 4 illustrates the average deviations between the numerical results and the experimental data, showing discrepancies of 7.6% in the heat transfer coefficient and 16.8% in the pressure drop. These differences are mainly due to geometric irregularities within the pin-fin array and the presence of solder meniscuses at the pin-fin solder joints, as pointed out by Fugmann et al. These irregularities are due to the limitations of the manual manufacturing process and have a particular impact on the increased pressure drop observed in the experimental results. Nevertheless, the comparative analysis of flow resistance and heat transfer performance, as shown in Figure 4, underscores the reliability of our numerical simulations.

## 3. Results and Discussion

### 3.1. Flow and Temperature Fields in Micro-Pin-Fin Arrays

**Figure 5:**
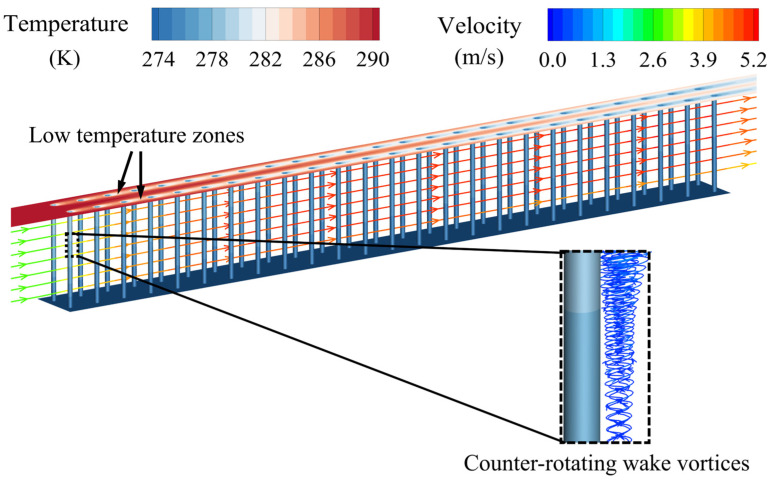
Three-dimensional streamlines and temperature distribution in the pin-fin array at *U_in_* = 3 m/s (*d* = 0.2 mm, *S_x_* = 1.3 mm, and *S_y_* = 1.2 mm). Streamlines are colored by velocity magnitude, with the wake vortices of the most upstream pin highlighted within the black dashed line.

Figure 5 shows the flow and temperature fields for a representative micro-pin-fin array with *d* = 0.2 mm, *S_x_* = 1.3 mm, and *S_y_* = 1.2 mm. The high-temperature airflow is blocked by the pins as it passes through the fin array, increasing the airflow velocity near the midplane between transverse adjacent pins with the constraint of mass conservation. The airflow temperature appreciably decreases after passing through the array due to the continuous heat transfer from the airflow to the pins and tube wall. At the rear of the pins, boundary layer separation occurs, forming a series of low-velocity recirculation zones characterized by pairs of counter-rotating wake vortices, which are crucial for the flow resistance of individual pins. As indicated by the black arrows in Figure 5, low-temperature zones, consisting of wake vortices with their downstream airflow, appear in the regions between streamwise adjacent pins, negatively affecting the heat transfer between the pins and the airflow, which is discussed in detail in Section 3.2 in relation to different pin pitches and diameters.

**Figure 6:**
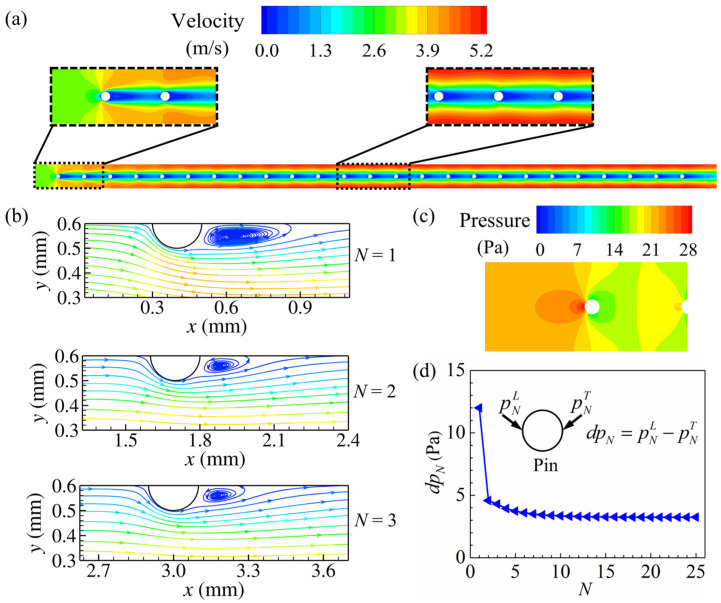
Evolution of airflow along the streamwise direction at *U_in_* = 3 m/s (*d* = 0.2 mm, *S_x_* = 1.3 mm, and *S_y_* = 1.2 mm): (**a**) velocity distribution at *z* = 4 mm; (**b**) streamlines near the first three pins at *z* = 4 mm, colored by velocity magnitude; (**c**) pressure distribution around the most upstream pin at *z* = 4 mm; (**d**) variation of the pressure difference between the leading and trailing edges of individual pin with the streamwise position number *N* of the pin.

The airflow velocity and pressure distribution vary significantly along the streamwise direction as the airflow passes through the first few pins of the array. For the most upstream pin, the velocity of the airflow decreases rapidly near the leading edge of the pin to form a high-pressure zone, while a low-pressure zone appears at its rear with the low-velocity wake vortices, as shown in Figure 6a–c. Further downstream, the airflow outside the wake vortices continuously flows into the region between streamwise adjacent pins along with the recovery of pressure. Since the airflow in front of the downstream pins has a lower velocity than that of the first pin, the wake vortices of the second and third pins are smaller, and these wake vortices are also more similar in size. The frontal airflow with lower velocities and the smaller-sized wake vortices result in smaller pressure differences between the leading and trailing edges of the second and third pins compared to the first pin, as shown in Figure 6d. It can be observed from Figure 6a,d that the velocity profile varies slightly along the flow direction as the airflow flows further downstream, and the similar flow structure around the pins causes the pressure difference *dp_N_* to gradually converge to a constant. Combining pin fins with different diameters and pitches, the flow structures at the streamwise position *x* ranging from 10.2 mm to 12.1 mm are selected for analysis in the following section due to their similarity to those observed downstream.

### 3.2. Effects of Streamwise and Transverse Pin Pitches

**Figure 7:**
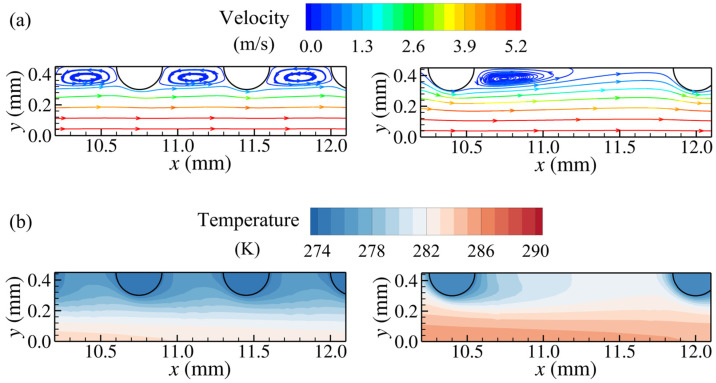
Streamlines and temperature distributions for pin fins with different streamwise pitches at *z* = 4 mm (*d* = 0.3 mm, *S_y_* = 0.9 mm, and *U_in_* = 3 m/s): (**a**) streamlines for *S_x_* = 0.7 mm (**left**) and *S_x_* = 1.6 mm (**right**); (**b**) temperature distributions for *S_x_* = 0.7 mm (**left**) and *S_x_* = 1.6 mm (**right**).

The effects of the streamwise and transverse pitches on the flow and heat transfer characteristics are elucidated in this section. Figure 7 shows the streamlines and temperature contours of the pin fins with different streamwise pitches at an inlet velocity of 3 m/s. For the pin fin with *d* = 0.3 mm, *S_x_* = 0.7 mm, and *S_y_* = 0.9 mm, the low-velocity and low-temperature wake vortices occupy the region between streamwise adjacent pins, resulting in lower temperature gradients on both the front and rear of the pins, with heat transfer from the airflow concentrating on the transverse sides of the pins. As the streamwise pitch increases to 1.6 mm, wake vortices with larger streamwise dimensions appear downstream of the pins, and part of the higher-velocity airflow outside the wake vortices can flow into the region between streamwise adjacent pins resulting in an increase in the temperature of these regions. It can be observed from Figure 7b that for the pin fin with a large streamwise pitch, higher temperature gradients appear at the front of the pins, with an enhanced heat transfer at the frontal sides of the pins. Meanwhile, unlike the pins with a streamwise pitch of 0.7 mm, whose frontal and rear sides are adjacent to low-pressure wake vortices, the pins with a streamwise pitch of 1.6 mm experience larger pressure differences between their frontal and rear sides since the higher-velocity flow from the outside of the wake vortices can impinge on the frontal sides of the pins, implying a larger flow resistance of individual pins.

**Figure 8:**
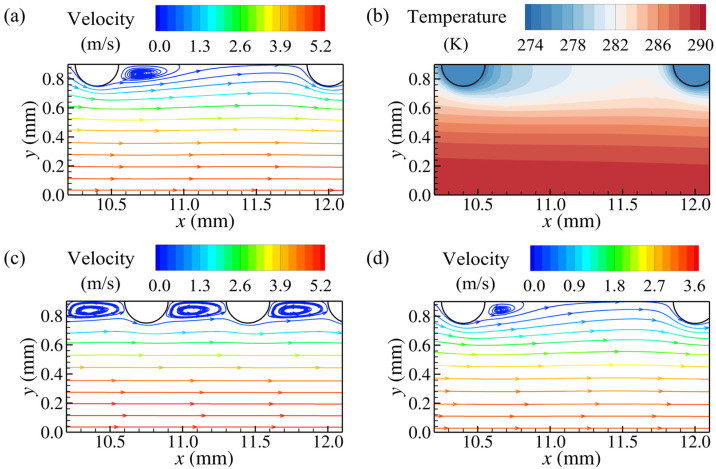
Temperature distribution and streamlines for pin fins with different streamwise pitches at *z* = 4 mm (*d* = 0.3 mm, *S_y_* = 1.8 mm): (**a**) streamlines for *S_x_* = 1.6 mm and *U_in_* = 3 m/s; (**b**) temperature distribution for *S_x_* = 1.6 mm and *U_in_* = 3 m/s; (**c**) streamlines for *S_x_* = 0.7 mm and *U_in_* = 3 m/s; (**d**) streamlines for *S_x_* = 1.6 mm and *U_in_* = 2 m/s. The scale magnitude of the legend in (**d**) is adjusted to adapt to the variation of the airflow velocity.

The transverse pin pitch also has a significant effect on the flow and temperature fields within the pin-fin arrays. Comparing Figure 7 and Figure 8, it can be seen that the airflow velocity near the pins with a wider transverse pitch is lower than that for pins with a narrower transverse pitch. As the transverse pitch increases from 0.9 mm to 1.8 mm, for pins with *d* = 0.3 mm and *S_x_* = 1.6 mm, the recirculation zones at their rear are reduced in size, attributed to lower airflow velocities around the pins. However, for pins with *d* = 0.3 mm and *S_x_* = 0.7 mm, the low-velocity wake vortices still fill the regions between streamwise adjacent pins, with lower temperature gradients at the front of the downstream pins. Pins with a larger transverse pitch create weaker disturbances to the airflow due to the fewer pins in the same transverse length, and the airflow away from the pins in the transverse direction can maintain higher temperatures. In addition, Figure 8a,d show the streamlines for the pin fins with *d* = 0.3 mm, *S_x_* = 1.6 mm, and *S_y_* = 1.8 mm at the inlet velocities of 3 m/s and 2 m/s, respectively. As the inlet velocity decreases, the ability of the boundary layer separation weakens and the size of the wake vortices around the pins decreases significantly, while the airflow evolution at other positions remains similar.

**Figure 9:**
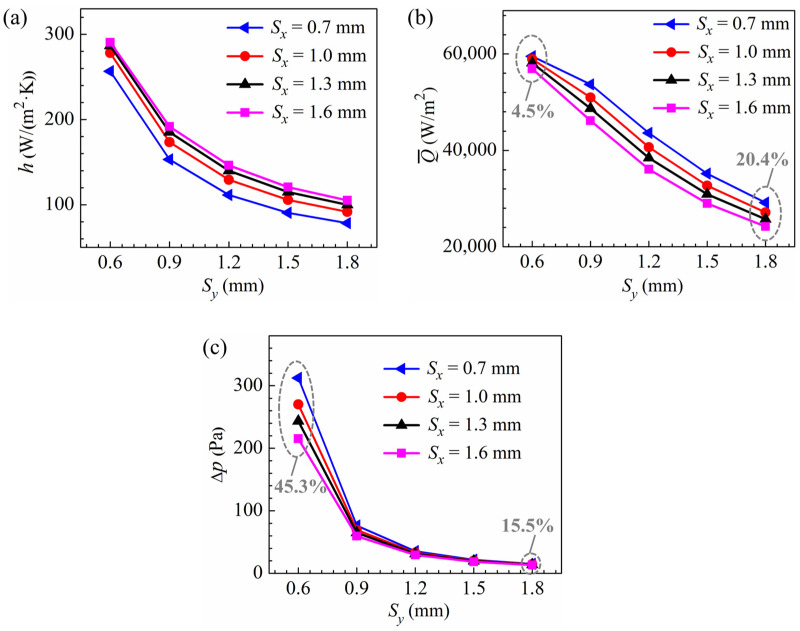
Performance comparison of pin fins with different pitches (*d* = 0.3 mm and *U_in_* = 3 m/s): (**a**) heat transfer coefficient; (**b**) heat transfer rate per unit frontal area; (**c**) pressure drop.

The effect of the transverse pin pitch on the heat transfer coefficient, heat transfer rate per unit frontal area, and pressure drop for the pin fins with a pin diameter of 0.3 mm is illustrated in Figure 9. The heat transfer coefficients increase as the transverse pitch decreases since the pin fins create stronger disturbances to the flow with a better mixing of the airflows of different temperatures, and the variations of the heat transfer coefficients with the transverse pitch are more pronounced at smaller pitches. For the pin fins with smaller transverse pitches, the heat transfer rates per unit frontal area are higher, owing to the larger heat transfer coefficients and increased heat transfer areas. However, the variations of $\overset{-}{Q}\;$ with the transverse pitch are not as significant as those of heat transfer coefficients at smaller pitches, as they are limited by reduced mean temperature differences between the airflow and the heating surface. In addition, with a smaller transverse pitch, both the higher resistance of individual pins, due to increased flow velocities near the pins, and the larger number of pins in the same frontal area result in a greater pressure drop. As shown in Figure 9c, the rate of the increase in pressure drop accelerates significantly at smaller pitches.

For the pin fins with a pin diameter of 0.3 mm, the heat transfer coefficients increase with larger streamwise pitch due to enhanced heat transfer at the frontal sides of the pins. The rate of increase in the heat transfer coefficient is substantially reduced when the streamwise pitch is large enough, as limited by the reduced number of pins. For pin fins with smaller streamwise pitches, despite lower heat transfer coefficients, $\overset{-}{Q}$ is higher as a result of the larger heat transfer area. The variations of $\overset{-}{Q}$ with streamwise pitch become insignificant at small enough transverse pitches. As shown in Figure 9b, at an inlet velocity of 3 m/s, the increase in $\overset{-}{Q}$ is 20.4% for the fins with a transverse pitch of 1.8 mm as the streamwise pitch is reduced from 1.6 mm to 0.7 mm, while the increase is only 4.5% for the fins with a transverse pitch of 0.6 mm. Decreasing the streamwise pitch reduces the flow resistance of individual pins since the region between streamwise adjacent pins is increasingly occupied by wake vortices. However, the total pressure losses for pin fins with smaller streamwise pitches are higher due to the increased number of pins. The differences in pressure drops among fins with different streamwise pitches become significantly larger with decreasing transverse pitch. Specifically, at an inlet velocity of 3 m/s, the increase in pressure drop is 15.5% for the pin fins with a transverse pitch of 1.8 mm but 45.3% for the pin fins with a transverse pitch of 0.6 mm as the streamwise pitch decreases from 1.6 mm to 0.7 mm. For the other calculated velocities, i.e., 1.5 m/s, 2 m/s, and 2.5 m/s, the streamwise and transverse pin pitches have similar effects on the flow and heat transfer characteristics of the pin-fin configurations with a pin diameter of 0.3 mm.

**Figure 10:**
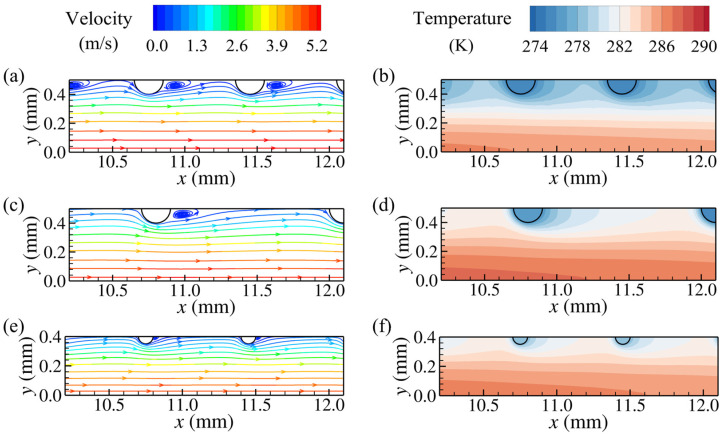
Streamlines and temperature distributions for different pin-fin configurations at *z* = 4 mm with *U_in_* = 3 m/s: (**a**,**b**): streamlines and temperature distribution for *S_x_* = 0.7 mm, *S_y_* = 1.0 mm, and *d* = 0.2 mm, respectively; (**c**,**d**): streamlines and temperature distribution for *S_x_* = 1.3 mm, *S_y_* = 1.0 mm, and *d* = 0.2 mm, respectively; (**e**,**f**) streamlines and temperature distribution for *S_x_* = 0.7 mm, *S_y_* = 0.8 mm, and *d* = 0.1 mm, respectively.

The size of the recirculation zone at the rear of the pin decreases with a reduction in diameter. As shown in Figure 10, for the pin fins with pin diameters of 0.1 mm and 0.2 mm, even at the minimum streamwise pitch, i.e., 0.7 mm, the regions between streamwise adjacent pins are not filled with wake vortices. As the streamwise pitch increases, the interference from the wake vortices of the pins with a diameter of 0.2 mm on the downstream adjacent ones decreases, resulting in significantly increased temperature gradients on the front of the downstream pins, similar to those for the pins with a diameter of 0.3 mm. For the pin fins with a diameter of 0.1 mm, all the streamwise pitches in Table 1 are much larger than the sizes of the wake vortices, with weaker thermal interference between streamwise adjacent pins, as depicted in Figure 10e,f. Increasing the streamwise pitch has a less significant effect on improving the heat transfer on the frontal sides of the pins with a diameter of 0.1 mm compared to the larger-diameter pins. The effects of the transverse pitch on the flow and temperature fields for the pin fins with diameters of 0.1 mm and 0.2 mm are similar to those observed for the 0.3 mm diameter pin fins.

**Figure 11:**
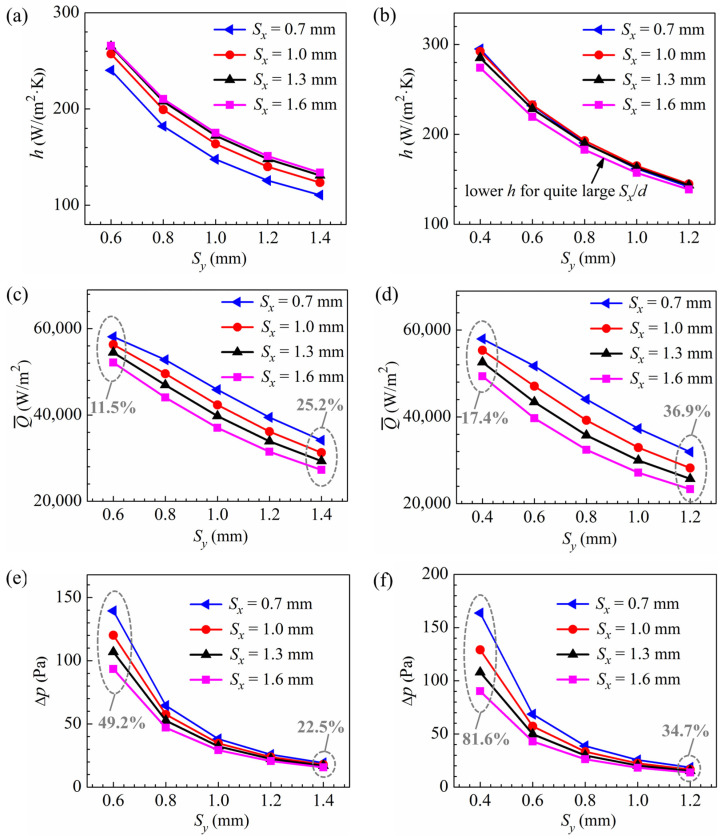
Performance comparison of pin fins with different pitches at *U_in_* = 3 m/s: (**a**) heat transfer coefficient for *d* = 0.2 mm; (**b**) heat transfer coefficient for *d* = 0.1 mm; (**c**) heat transfer rate per unit frontal area for *d* = 0.2 mm; (**d**) heat transfer rate per unit frontal area for *d* = 0.1 mm; (**e**) pressure drop for *d* = 0.2 mm; (**f**) pressure drop for *d* = 0.1 mm.

Figure 11 shows the effects of pin pitches on the heat transfer coefficient, heat transfer rate per unit frontal area, and pressure drop for the pin fins with smaller diameters at an inlet velocity of 3 m/s. For the other three velocities, the effects of pin pitches on the hydraulic and thermal performance are similar, with reduced values for all performance metrics. As depicted in Figure 11, the heat transfer coefficient, heat transfer rate $\overset{-}{Q}$, and pressure drop all increase with decreasing transverse pitch for the pin fins with pin diameters of 0.2 mm and 0.1 mm, with larger increase rates in the heat transfer coefficient and pressure drop at smaller transverse pitches, consistent with those for the 0.3 mm diameter pin fins.

As the streamwise pitch increases from 0.7 mm to 1.6 mm, the heat transfer coefficients tend to be greater for the pin fins with a pin diameter of 0.2 mm, with smaller differences at higher streamwise pitches, similar to the case for the pin fins with a pin diameter of 0.3 mm. However, for the pin fins with a pin diameter of 0.1 mm, the advantage of enhanced heat transfer at the frontal sides of the pins makes it difficult to offset the disadvantage of reduced pin numbers, especially at a streamwise pitch of 1.6 mm, where the streamwise pitch is 16 times the diameter and the heat transfer coefficients are noticeably lower compared to the other fins. The heat transfer rate $\overset{-}{Q}$ and pressure drop both increase as the streamwise pitch decreases, with the variation in pressure drop becoming more remarkable at smaller transverse pitches. As shown in Figure 11c–f, for the pin fins with diameters of 0.2 mm and 0.1 mm at an inlet velocity of 3 m/s, when the streamwise pitch decreases from 1.6 mm to 0.7 mm, the pressure drop at the maximum transverse pitch increases by 22.5% and 34.7%, respectively, with increases in $\overset{-}{Q}$ by 25.2% and 36.9%, respectively, while at the minimum transverse pitch, the pressure drop increases by 49.2% and 81.6%, respectively, with increases in $\overset{-}{Q}$ by only 11.5% and 17.4%, respectively. The increases in pressure drops are significantly higher than those of heat transfer rates for the pin fins with small-enough transverse pitches.

Our results indicate that while the heat transfer rate $\;\overset{-}{Q}$ improves with reductions in both streamwise and transverse pin pitches, the pressure drop increases with smaller pitches. This suggests that using pin-fin arrays with reduced pitches may not optimize overall efficiency in practical applications, since both the pressure drop and heat transfer rate increase simultaneously. This is especially true when the transverse pitch is significantly reduced, as further reductions in both the streamwise and transverse pitches result in significant increases in the pressure drop, while the gains in $\overset{-}{Q}$ remain relatively modest. In such scenarios, the economic feasibility of improving heat transfer by reducing the pin pitch is questionable. To strike a balance between heat transfer improvement and flow resistance, we compared the heat transfer rate $\overset{-}{Q}$ achieved at the same fan power $\overset{-}{P}$ to evaluate the overall performance of different fin configurations.

**Figure 12:**
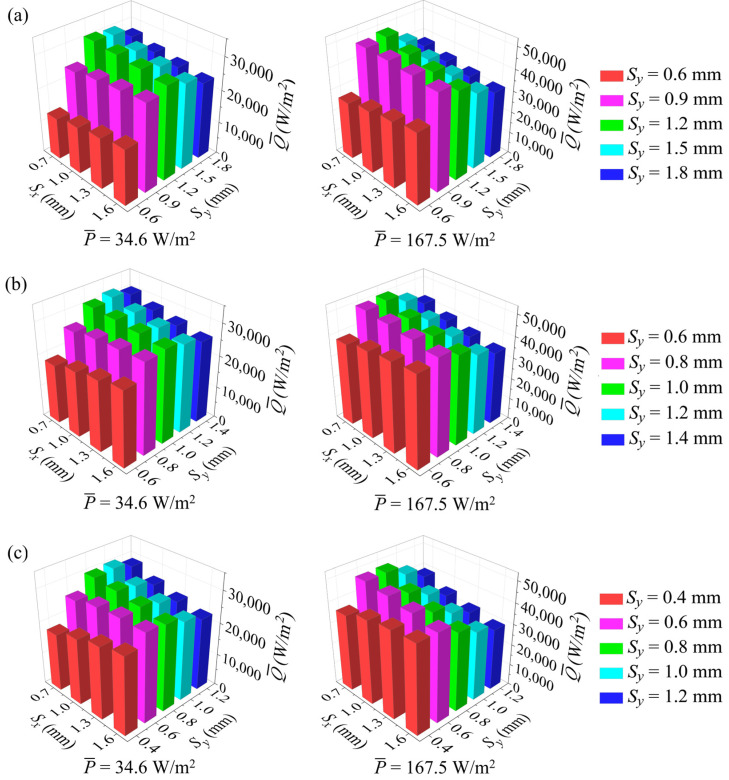
Variations of heat transfer rate $\overset{-}{Q}$ with pin pitches at $\overset{-}{P}$ = 34.6 W/m^2^ (**left**) and 167.5 W/m^2^ (**right**): (**a**) pin fins with *d* = 0.3 mm; (**b**) pin fins with *d* = 0.2 mm; (**c**) pin fins with *d* = 0.1 mm.

The fan powers per unit frontal area consumed by LF-1 at inlet velocities of 1.5 m/s, 2 m/s, 2.5 m/s, and 3 m/s were selected as benchmarks. These values are also used in the following section to compare the overall performance of the pin fins and louvered fins, which are 34.6 W/m^2^, 66.3 W/m^2^, 110.2 W/m^2^, and 167.5 W/m^2^, respectively. Figure 12 presents $\overset{-}{Q}$ for pin fins with different pin pitches and pin diameters at the lowest and highest power levels. As the transverse pitch decreases from a larger value, $\overset{-}{Q}$ reaches a peak and then decreases at smaller transverse pitches as a result of the rapid increase in the pressure drop, indicating that an optimal transverse pitch is available for the pin fins. As shown in Figure 12, the optimal transverse pitch tends to be smaller with increasing fan power and streamwise pitch. For example, for the pin fins with a pin diameter of 0.2 mm, as the streamwise pitch increases from 0.7 mm to 1.6 mm, the optimal transverse pitch decreases from 1.2 mm to 1.0 mm at the lowest power level; then, it further decreases to 0.8 mm as $\overset{-}{P}$ increases to 167.5 W/m^2^.

For the pin fins with larger transverse pitches, $\overset{-}{Q}$ decreases with increasing streamwise pitch at the equivalent fan power level. However, for a sufficiently small transverse pitch, the larger streamwise pitch contributes to enhancing the overall performance of the pin fins, with a greater $\overset{-}{Q}$ for increasing streamwise pitch. The effects of streamwise and transverse pitches on pin fins of different diameters are similar, and the optimal transverse pitch tends to be smaller as the diameter decreases. It can also be observed from Figure 12 that for pin fins with the same pitch, if both the streamwise and transverse pitches are sufficiently small, smaller-diameter pin fins exhibit a higher $\;\overset{-}{Q}$. For instance, for *S_x_* = 0.7 mm and *S_y_* = 0.6 mm, the pin fin with a 0.1 mm diameter has the highest $\;\overset{-}{Q}$, while the pin fin with a 0.3 mm diameter has the lowest. As the streamwise and transverse pitches increase, the larger-diameter pin fins gradually demonstrate an advantage in heat transfer. In addition, at higher fan power levels, larger-diameter pin fins also tend to perform better in heat transfer.

By integrating $\overset{-}{Q}\;$ for all four fan powers, the optimal pin-fin configurations for the three diameters are identified, with the streamwise pitch of 0.7 mm for all cases and the transverse pitches of 0.8 mm, 1.0 mm, and 1.2 mm for pin diameters of 0.1 mm, 0.2 mm, and 0.3 mm, respectively, named PF-1, PF-2, and PF-3, respectively. Interestingly, the ratio of transverse pitch to diameter is not constant for the optimal configurations but increases for smaller-diameter pin fins.

### 3.3. Comparison of the Overall Performance between Pin Fins and Louvered Fins

**Figure 13:**
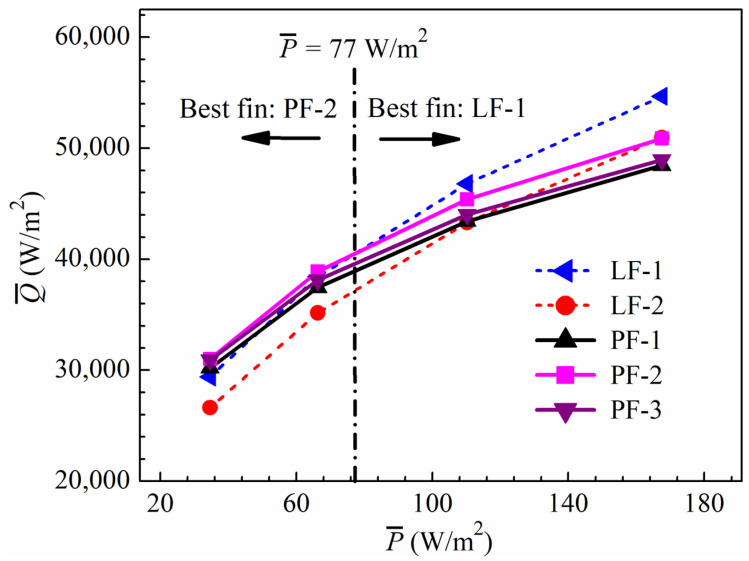
Comparison of heat transfer rate $\overset{-}{Q}$ among different fin configurations.

In this section, the optimal pin-fin configurations are compared with the louvered fins LF-1 and LF-2. As shown in Figure 13, PF-2 and PF-1 exhibit the best and worst performance among the three optimal configurations, respectively, and the former has a heat transfer performance comparable to the louvered fins. At the lowest power level, the $\overset{-}{Q}$s of all three pin fins are higher than those of the louvered fins, with the pin-fin PF-2 increasing by 5.5% and 16.5% compared to LF-1 and LF-2, respectively. The advantage of pin fins over louvered fins in heat transfer gradually disappears as the fan power increases. When $\;\overset{-}{P}$ is around 77 W/m^2^, LF-1 replaces PF-2 as the configuration with the best overall performance among the five fin designs. At the highest power level, the $\overset{-}{Q}$ of PF-2 is 6.9% lower than that of LF-1 but equivalent to that of the more compact one, LF-2. In addition, PF-2 consumes fewer fins compared to louvered fins for the same frontal area, with its fin volume being only 63.6% of that of the LF-1. Considering its satisfactory heat transfer efficiency, with a more uniform fin structure for drainage and reduced fin consumption compared to the louvered fins, the micro-pin-fin PF-2 is recommended for the PFFTHXs used as evaporators. 

## 4. Conclusions

Three-dimensional numerical simulations were performed to study the hydraulic and thermal performance of PFFTHXs with a variety of pin-fin configurations. This study used 60 configurations across three pin diameters to investigate the influence of pin pitch on the effectiveness of the heat transfer enhancement using micro pin fins. Heat transfer rates per unit frontal area were measured at four equivalent fan power levels, allowing the performance of various pin-fin configurations to be evaluated relative to two types of louvered fins. The main findings can be summarized as follows:

(1) Pin pitch significantly affects the airflow near the pin and the local heat transfer capacity. Increasing the streamwise pin pitch reduces the disturbance from low-velocity wake vortices caused by boundary layer separation on downstream pins, thereby increasing the heat transfer in front of these pins due to larger local temperature gradients.
(2) For all pin diameters, reducing the streamwise and transverse pin pitch increases both the heat transfer rate per unit frontal area and the pressure drop under constant flow velocity conditions. Notably, the variation in pressure drop is more pronounced at smaller transverse pitches, indicating a decrease in overall performance and suggesting that smaller streamwise pitches are less effective under these conditions.
(3) Optimal configurations for micro pin fins with diameters of 0.1 mm, 0.2 mm, and 0.3 mm were identified at the four fan power levels. These optimal configurations are characterized by a uniform streamwise pitch of 0.7 mm and varying transverse pitches of 0.8 mm, 1.0 mm, and 1.2 mm, respectively, with the ratio of transverse pitch to pin diameter increasing as the pin diameter decreases.
(4) Among the optimal configurations, the 0.2 mm pin-diameter configuration consistently outperformed the other diameters at all fan power levels. Compared to louvered fins, this configuration offers satisfactory heat transfer efficiency with lower fin consumption and a more uniform structure, making it particularly suitable for use in evaporator applications.

## Figures and Tables

**Table 1:** Geometric parameters of different pin fins.

*d* (mm)	*F_h_* (mm)	*F_d_* (mm)	*S_x_* (mm)	*S_y_* (mm)
0.1	8	32	0.7, 1.0, 1.3, 1.6	0.4, 0.6, 0.8, 1.0, 1.2
0.2	8	32	0.7, 1.0, 1.3, 1.6	0.6, 0.8, 1.0, 1.2, 1.4
0.3	8	32	0.7, 1.0, 1.3, 1.6	0.6, 0.9, 1.2, 1.5, 1.8

**Table 2:** The thermophysical properties of the air and aluminum pin fins.

Type	Parameter	Value
Air	Thermal conductivity	0.0247 W/(m·K)
	Specific heat	1007 J/(kg·K)
	Density	1.225 kg/m^3^
	Dynamic viscosity	1.8 × 10^–5^ kg/(m·s)
Aluminum fin	Thermal conductivity	202.4 W/(m·K)

## Data Availability

The original contributions presented in the study are included in the article, further inquiries can be directed to the corresponding authors.

## Nomenclature

*A*	area, m^2^
*A_o_*	total heat transfer area, m^2^
*c_p_*	specific heat, J/(kg·K)
*d*	pin diameter, m
*dp*	pressure difference between the leading and trailing edges of a pin, Pa
*F_d_*	flow depth, m
*F_h_*	fin height, m
*F_p_*	fin pitch of the louvered fin, m
*h*	heat transfer coefficient, W/(m^2^·K)
*k*	thermal conductivity, W/(m·K)
$\overset{-}{P}$	fan power per unit frontal area, W/m^2^
*p*	pressure, Pa
Δ*p*	pressure drop, Pa
$\overset{-}{Q}$	heat transfer rate per unit frontal area, W/m^2^
*Q*	heat transfer rate, W
*Re*	Reynolds number
*S_x_*	streamwise pin pitch, m
*S_y_*	transverse pin pitch, m
Δ*s*	size of the first layer element on fin surface, m
*T*	temperature, K
Δ*T_m_*	logarithmic mean temperature difference, K
*T_w_*	tube wall temperature, K
*U*	velocity, m/s
***u***	velocity vector, m/s
Subscripts	
*a*	air
*c*	cross-section with minimal flow area
*in*	inlet
*N*	streamwise position number
*out*	outlet
*f*	fin
Superscripts	
*L*	leading edge of the pin
*T*	trailing edge of the pin
Greek symbols	
*μ*	dynamic viscosity, kg/(m·s)
*ρ*	density, kg/m^3^
Abbreviations	
CFD	computational fluid dynamics
LFFTHXs	louvered-fin and flat-tube heat exchangers
PFFTHXs	pin-fin and flat-tube heat exchangers

## References

[1] Qi Z. (2013). Water retention and drainage on air side of heat exchangers—A review. Renew. Sustain. Energy Rev..

[2] Xiong T., Ying Y., Han B., Yan G., Yu J. (2021). Comparison of energy supplies and consumptions in heat pump systems using finned tube and microchannel heat exchangers during defrosting. Int. J. Refrig..

[3] Effendy M., Yao Y., Yao J., Marchant D.R. Pin-fin shape and orientation effects on wall heat transfer predictions of gas turbine blade. Proceedings of the 5th International Conference on Engineering, Technology, and Industrial Application (ICETIA) 2018.

[4] Xie G.N., Sundén B., Utriainen E., Wang L. (2010). Computational Analysis of Pin-Fin Arrays Effects on Internal Heat Transfer Enhancement of a Blade Tip Wall. J. Heat Transf. Trans. ASME.

[5] Yan Y., Zhao T., He Z., Yang Z., Zhang L. (2021). Numerical investigation on the characteristics of flow and heat transfer enhancement by micro pin-fin array heat sink with fin-shaped strips. Chem. Eng. Process..

[6] Markowski P.M., Gierczak M., Dziedzic A. (2019). Modelling of the temperature difference sensors to control the temperature distribution in processor heat sink. Micromachines.

[7] Manjunath M.S., Karanth K.V., Sharma N.Y. (2019). Numerical Analysis of Flat Plate Solar Air Heater Integrated With an Array of Pin Fins on Absorber Plate for Enhancement in Thermal Performance. J. Sol. Energy Eng. Trans.-ASME.

[8] Arunkumar H., Kumar S., Karanth K.V. (2020). Analysis of a solar air heater for augmented thermohydraulic performance using helicoidal spring shaped fins-A numerical study. Renew. Energy.

[9] Pandit J., Thompson M., Ekkad S.V., Huxtable S.T. (2014). Effect of pin fin to channel height ratio and pin fin geometry on heat transfer performance for flow in rectangular channels. Int. J. Heat Mass Transf..

[10] Arie M., Hymas D., Singer F., Shooshtari A., Ohadi M. Performance characterization of a novel cross-media composite heat exchanger for air-to-liquid applications. Proceedings of the 2019 18th IEEE Intersociety Conference on Thermal and Thermomechanical Phenomena in Electronic Systems (ITherm).

[11] Arie M., Hymas D., Singer F., Shooshtari A., Ohadi M. (2020). An additively manufactured novel polymer composite heat exchanger for dry cooling applications. Int. J. Heat Mass Transf..

[12] Fugmann H., Di Lauro P., Sawant A., Schnabel L. (2018). Development of Heat Transfer Surface Area Enhancements: A Test Facility for New Heat Exchanger Designs. Energies.

[13] Sahiti N. (2015). Interrelation between pin length and heat exchanger performance. Appl. Therm. Eng..

[14] Ravanji A., Lee A., Mohammadpour J., Cheng S. (2023). Critical review on thermohydraulic performance enhancement in channel flows: A comparative study of pin fins. Renew. Sustain. Energy Rev..

[15] Bhandari P., Rawat K.S., Prajapati Y.K., Padalia D., Ranakoti L., Singh T. (2023). Design modifications in micro pin fin configuration of microchannel heat sink for single phase liquid flow: A review. J. Energy Storage.

[16] Xu J., Yao J., Su P., Lei J., Wu J., Gao T. Heat transfer and pressure loss characteristics of pin-fins with different shapes in a wide channel. Proceedings of the Turbo Expo: Power for Land, Sea, and Air.

[17] Choudhary V., Kumar M., Patil A.K. (2019). Experimental investigation of enhanced performance of pin fin heat sink with wings. Appl. Therm. Eng..

[18] Narato P., Wae-hayee M., Kaewchoothong N., Nuntadusit C. (2021). Heat transfer enhancement and flow characteristics in a rectangular channel having inclined pin arrays mounted on the endwall surface. Int. Commun. Heat Mass Transf..

[19] Kishore H., Pal M., Nirala C.K., Agrawal A. (2024). Thermal performance evaluation of Micro pin–fin heat exchangers: Part I—Geometrical Design parameters optimization. Int. J. Precis. Eng. Manuf..

[20] Kishore H., Pal M., Nirala C.K., Agrawal A. (2024). Thermal performance evaluation of Micro pin–fin heat exchangers: Part II—Numerical Simulation and Fabrication demonstration. Int. J. Precis. Eng. Manuf..

[21] Zhang J., Wu J., Xie Z., Lu Z., Guan X., Ge Y. (2023). Suitability of Embedded Liquid Cooling and Heat Generation for Chips. Micromachines.

[22] Li P., Fan X., Chen Z. (2016). Numerical study on the heat transfer of micro elliptic pin fins in a rectangular minichannel. Numer. Heat Transf. A-Appl..

[23] Fugmann H., Oltersdorf T., Schnabel L. Metal Wire Structures as Heat Transfer Surface Area Enlargement—Design Study and Potential Analysis for Air-to-Water Heat Pumps. Proceedings of the 12th IEA Heat Pump Conference.

[24] Fugmann H., Schnabel L., Frohnapfel B. (2019). Heat transfer and pressure drop correlations for laminar flow in an in-line and staggered array of circular cylinders. Numer. Heat Transf. A-Appl..

[25] Jin W., Wu J., Jia N., Lei J., Ji W., Xie G. (2021). Effect of shape and distribution of pin-fins on the flow and heat transfer characteristics in the rectangular cooling channel. Int. J. Therm. Sci..

[26] Zhou Q., Wang H., Liu S., Wei H., Hu G. (2024). Assessment of the heat transfer efficiency of perforated louvered fins for improved drainage. Int. J. Heat Mass Transf..

[27] Saleem A., Kim M.-H. (2017). CFD analysis on the air-side thermal-hydraulic performance of multi-louvered fin heat exchangers at low Reynolds numbers. Energies.

[28] Saleem A., Kim M.-H. (2017). Air-side thermal hydraulic performance of microchannel heat exchangers with different fin configurations. Appl. Therm. Eng..

[29] Saleem A., Kim M.-H. (2022). Airside thermal performance of louvered fin flat-tube heat exchangers with different redirection louvers. Energies.

[30] Bergelin O., Brown G., Doberstein S. (1952). Heat transfer and fluid friction during flow across banks of tubes—IV: A study of the transition zone between viscous and turbulent flow. Trans. Am. Soc. Mech. Eng..

[31] Fugmann H., Laurenz E., Schnabel L. (2017). Wire Structure Heat Exchangers-New Designs for Efficient Heat Transfer. Energies.

[32] Kailkhura G., Mandel R.K., Shooshtari A., Ohadi M. (2022). Numerical and Experimental Study of a Novel Additively Manufactured Metal-Polymer Composite Heat-Exchanger for Liquid Cooling Electronics. Energies.

